# RNAseq by Total RNA Library Identifies Additional RNAs Compared to Poly(A) RNA Library

**DOI:** 10.1155/2015/862130

**Published:** 2015-10-12

**Authors:** Yan Guo, Shilin Zhao, Quanhu Sheng, Mingsheng Guo, Brian Lehmann, Jennifer Pietenpol, David C. Samuels, Yu Shyr

**Affiliations:** ^1^Center for Quantitative Sciences, Vanderbilt University, Nashville, TN 37232, USA; ^2^Department of Biochemistry, Vanderbilt University, Nashville, TN 37232, USA; ^3^Center for Human Genetics Research, Vanderbilt University, Nashville, TN 37232, USA

## Abstract

The most popular RNA library used for RNA sequencing is the poly(A) captured RNA library. This library captures RNA based on the presence of poly(A) tails at the 3′ end. Another type of RNA library for RNA sequencing is the total RNA library which differs from the poly(A) library by capture method and price. The total RNA library costs more and its capture of RNA is not dependent on the presence of poly(A) tails. In practice, only ribosomal RNAs and small RNAs are washed out in the total RNA library preparation. To evaluate the ability of detecting RNA for both RNA libraries we designed a study using RNA sequencing data of the same two breast cancer cell lines from both RNA libraries. We found that the RNA expression values captured by both RNA libraries were highly correlated. However, the number of RNAs captured was significantly higher for the total RNA library. Furthermore, we identify several subsets of protein coding RNAs that were not captured efficiently by the poly(A) library. One of the most noticeable is the histone-encode genes, which lack the poly(A) tail.

## 1. Introduction

With the advancement of high throughput sequencing technology, advanced data mining techniques have been developed for high throughput DNA sequencing data [1, 2]. Similar data mining techniques can be applied to RNAseq data. RNAseq technology can be categorized into three subclasses by the types of RNA sequenced: messenger RNA (mRNA or protein coding RNA), micro RNA (miRNA), and total RNA. The sequencing method is the same but each differs in the RNA species present for cDNA synthesis and subsequent library construction. The cDNA library for mRNAseq is made only from the poly(A) mRNA. Small RNAs are not captured during oligo-dT based mRNA enrichment. To date, the most popular application of RNAseq technology is mRNA sequencing because most researchers use RNAseq as a replacement for microarray to perform high throughput gene expression profiling [3–6] and coding regions remain the focus of human disease research.

Long noncoding RNA (lncRNA), on the other hand, was traditionally believed to be nonfunctional. However, many recent studies have shown evidence for the functionality of lncRNA [7, 8], such as roles in high-order chromosomal dynamics [9], embryonic stem cell differentiation [10], telomere biology [11], subcellular structural organization [12], and breast cancer [13, 14]. The interest in lncRNA grew considerably as the evidence of lncRNA's role in various biological contexts accumulated in the recent years. LncRNAs are usually defined as noncoding RNA with length more than 200 base pairs [7, 15]. Structurally, lncRNAs and mRNAs are very similar, as both can exhibit polyadenylation (poly(A)). The number of definable lncRNAs varies by study. An early study in 2007 estimated that there are 4 times more lncRNAs than protein coding RNA [16]. Another study claims to have identified 35,000 lncRNAs [17], and many of them have characteristics similar to mRNA such as 5′ capping, splicing, and polyadenylation, with the exception of open reading frames [17]. In the latest effort to quantify human lncRNA, the Encyclopedia of DNA Elements (ENCODE) [18] project identified 13,333 lncRNAs and further categorized them into four subclasses: (1) antisense, (2) large intergenic noncoding RNAs (lincRNA), (3) sense intronic, and (4) processed transcripts.

While it is possible to study lncRNAs using traditional microarrays, RNAseq has been proven to be the superior technology for this purpose due to its greater sensitivity and the ability to detect novel lncRNAs [19, 20]. The rise in the popularity and affordability of RNAseq technology is primarily responsible for the growing interest in and understanding of lncRNAs as researchers explore the presence of these stowaways in their mRNA data sets. In mRNA sequencing, mRNAs are captured based on the presence of a poly(A) tail. LncRNAs can also be captured provided they have a poly(A) tail. According to a study in 2005, it is estimated that 40% of lncRNA transcripts are nonpolyadenylated [21]. An alternative library preparation method for studying lncRNA is the total RNA library. Only ribosomal RNA is removed leaving small RNAs, mRNAs, and all forms of lncRNAs. This library preparation method is the most inclusive of RNA species but requires more sequencing reads due to the multiple RNA species present in the library, and ribosomal RNA reduction does not completely remove ribosomal RNA from the library due to their high abundance.

Total RNA sequencing theoretically should detect more lncRNAs due to its RNA selection independent of the poly(A) tail. However, total RNAseq costs more than mRNA sequencing (mRNA $500 versus total RNA $650) and the question of how many more lncRNAs does total RNA sequencing capture compared to mRNA sequencing has not been answered. Moreover, whether the mRNAs captured in total RNA sequencing are comparable to mRNA sequencing also remains unknown. To answer these questions, we designed the following study. We hypothesized that total RNA sequencing generates more relevant data than mRNA sequencing for the purpose of lncRNA research. Total RNA and mRNA libraries of two breast cancer cell line samples were built and sequenced. We analyzed the sequencing data and compared their usability for lncRNA and mRNA research.

## 2. Methods

Total RNAseq on two breast cancer cell lines HS578T and BT549 was performed by the Vanderbilt Technologies for Advanced Genomics (VANTAGE) core. Total RNA was isolated with the Aurum Total RNA Mini Kit. All samples were quantified on the QuBit RNA assay. RNA quality was checked using Agilent Bioanalyzer. RNA integrity number (RIN) for both samples was 10. RNAseq data was obtained by first using the Ribo-Zero Magnetic Gold Kit (human/mouse/rat) (Epicentre) to perform ribosomal reduction on 1 *μ*g total RNA following the manufacturer's protocol. After ribosomal RNA (rRNA) depletion, samples were then purified using the Agencourt RNAClean XP Kit (Beckman Coulter) according to the Epicentre protocol specifications. After purification, samples were eluted in 11 *μ*L RNase-free water. Next, 1 *μ*L ribosomal depleted samples were run on the Agilent RNA 6000 Pico Chip to confirm rRNA removal. After confirmation of rRNA removal, 8.5 *μ*L rRNA-depleted sample was input into the Illumina TruSeq Stranded RNA Sample Preparation kit (Illumina) for library preparation. The libraries were sequenced on Illumina High HiSeq 2500 with paired-end 100 base pair long reads. Raw RNAseq sequencing data generated from the poly(A) library of the same two cell lines were downloaded from the Gene Expression Omnibus (GEO) (GSM1172877: 19.8 million reads and GSM1172855: 15.3 million reads) for comparative purpose. The poly(A) libraries were prepared using Illumina TruSeq RNA Sample Preparation kit. Poly(A) RNA was purified with oligo dT magnetic beads, and the poly(A) RNA was fragmented with divalent cations followed by reverse transcription into cDNA and ligation of Illumina paired-end oligo adapters to the cDNA fragments. More detail of poly(A) library construction can be found at GEO website.

The raw data quality was examined using QC3 [22]. Alignment against human genome reference HG19 was performed using TopHat2 [23]. Novel gene quantification was performed using Cufflinks [24]. Additional quality control was carried out at alignment level based on the alignment quality control concept described in [25]. ENSEMBL gene transfer format (GTF) version GRCh37.35 was used to annotate the gene expression. We categorized the RNA into three subclasses: protein coding RNA, lncRNA, and other RNAs. This GTF contains 20327 protein coding RNAs, 13346 lncRNAs, and 24100 other RNAs (such as pseudogene and antisense). Read count per RNA was computed using HTSeq [26]. To avoid variation caused by total reads sequenced, raw read counts were normalized to the total read count by sample. Log2 transformations were performed on normalized read counts. To avoid log of zeroes, all read counts were increased by 1 before taking the log transformation. Differential expression analyses and additional quality control were conducted between poly(A) capture method and total RNA method using MultiRankSeq [27] which embeds three different RNAseq differential expression analysis methods: DESeq2 [28], edgeR [29], and baySeq [30]. DEseq2's results were selected for further analysis due to its ability to take paired samples into consideration. Cluster analysis was performed using Heatmap3 [31]. Functional analysis was carried out using gene set enrichment analysis (GSEA) [32], and gene ontology (GO) analysis was conducted using WebGestalt [33].

## 3. Results

**Figure 1:**
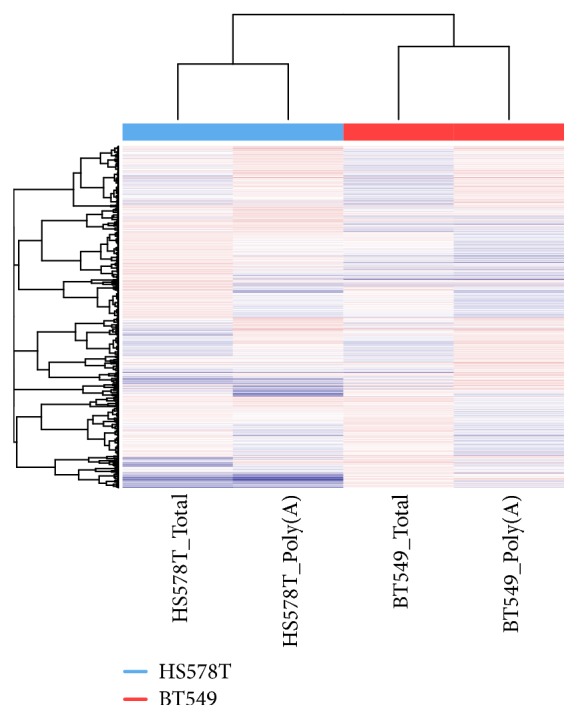
Cluster results of the two breast cancer cell lines. The poly(A) and total RNA libraries were constructed and sequenced by separated facilities. The samples clustered together by cell line type rather than library type or sequencing facility, which suggests that there is no severe heterogeneity of cell line and batch effect between sequencing.

Even though the RNAseq data were generated from the same cell lines, there could be potential heterogeneity and batch effect because the cell lines were cultured at two different labs and sequenced at two different facilities. To test if there is potential heterogeneity and batch effect, we conducted a cluster analysis using Heatmap3 [31]. Unsupervised cluster results showed cluster of cell line type rather than sequencing batch (Figure 1) which suggested that the RNAseq data of these two cell lines were similar; no severe heterogeneity and batch effect were observed.

The sequencing data went through rigorous quality control. To account for variation in number of reads sequenced within the 4 samples, read counts were adjusted by normalizing the total read count of each sample. In terms of proportion of reads mapped to lncRNA, total RNA library samples (3.62% and 3.23%) had a higher proportion than poly(A) library samples (0.85% and 1.02%). For protein coding RNA, poly(A) library samples (96.34% and 95.38%) mapped a higher proportion of reads than total RNA samples (92.47% and 93.45%). For other species of RNAs, poly(A) library samples (2.81% and 3.59%) and total RNA library samples (3.91% and 3.32%) had similar proportion of reads aligned.

**Figure 2:**
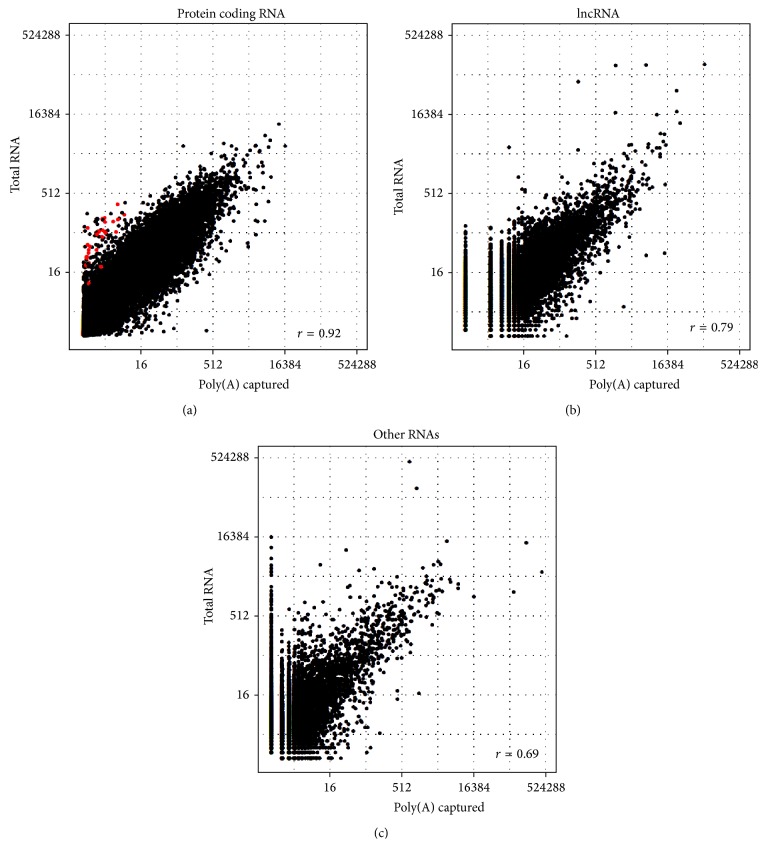
RNA expression level consistency between poly(A) and total RNA library samples. Read counts were normalized by total read count per sample and log2 transformed. (a) Consistency of expression of protein coding RNAs. The red color indicates histone-encoding genes. (b) Consistency of expression of lncRNAs. (c) Consistency of expression of other RNAs.

The distributions of read normalized read counts for protein coding RNA, lncRNA, and other RNAs can be seen in Figure S1 (in Supplementary Material available online at http://dx.doi.org/10.1155/2015/862130). All three types of RNAs were detected by both poly(A) and total RNA library building methods. To compare whether RNA expressions are comparable between the two RNA library building methods, we drew a scatter plot and computed their Pearson's correlation coefficients (Figure 2). All three types of RNA expression are highly agreeable between the two methods (protein coding RNA *r* = 0.92, lncRNA Pearson *r* = 0.79, and other RNAs *r* = 0.69). These results are consistent with previous findings [34] which suggest that RNA expression is consistently measured for poly(A) and total RNA sequencing library.

**Figure 3:**
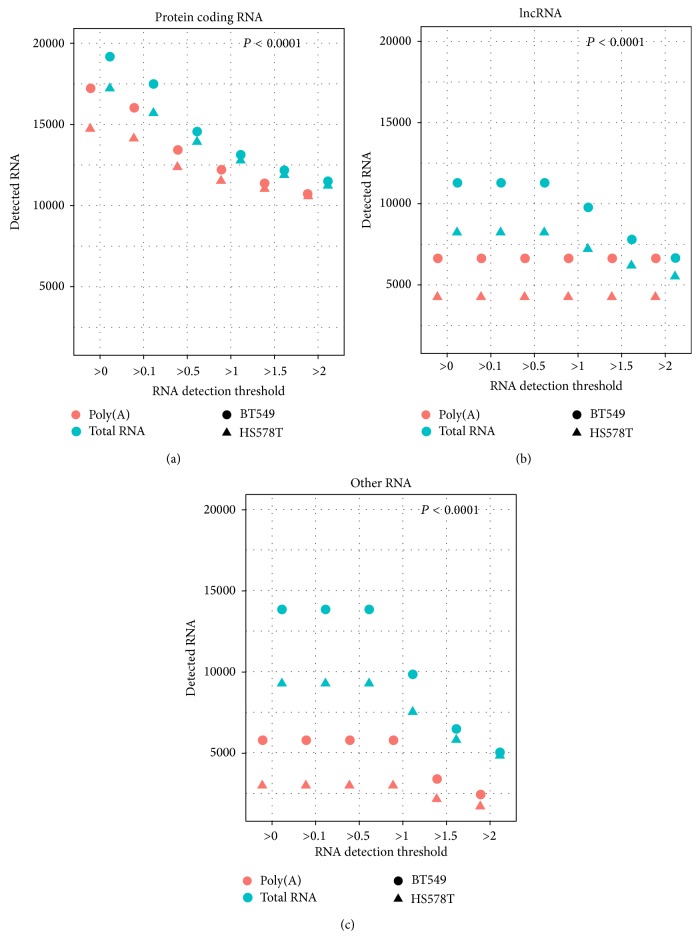
Number of RNAs detected at different detection thresholds for all three types of RNA. Total RNA library samples detected significantly more RNAs than poly(A) RNA library samples at all RNA detection thresholds. (a) Protein coding RNA. (b) lncRNA. (c) Other RNAs.

**Figure 4:**
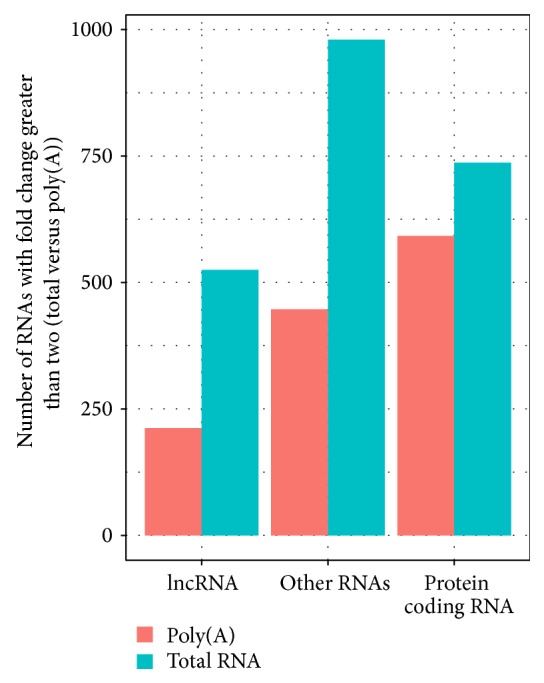
Using log2 fold change >2 as cutoffs, total RNA library samples had more RNAs with higher expression levels than poly(A) samples for all three types of RNAs.

Next, we examine the number of RNAs detectable by each library construction method. To determine whether RNA is detected, a cutoff value of the normalized read count was applied. Because this cutoff is arbitrary, we choose several different thresholds for sensitivity analysis. An RNA is considered detected if its normalized read count is above the detection threshold. We used the following thresholds: >0.1, >0.5, >1, >1.5, and >2. Regardless of which threshold we applied, samples from the total RNA method consistently showed higher numbers of RNAs detected for all three types of RNAs (Figure 3). This suggests that without the restriction of poly(A) selection, the total RNA library is capable of identifying more expressed RNAs (lncRNA *t*-test *P* < 0.0001, protein coding RNA *t*-test *P* < 0.0001, and other RNAs *t*-test *P* < 0.0001). Furthermore, we compared the number of genes that are differentially expressed between the two libraries' construction methods and found there were much higher expressed RNAs (log2 fold change > 2) for total RNA library samples than poly(A) library samples (Figure 4). We also counted the potential novel transcripts identified from Cufflinks. The two poly(A) library samples detected 4122 and 6169 potential new transcripts, and the two total RNA samples detected 53282 and 58111 potential new transcripts, roughly a 10-fold increase.

**Figure 5:**
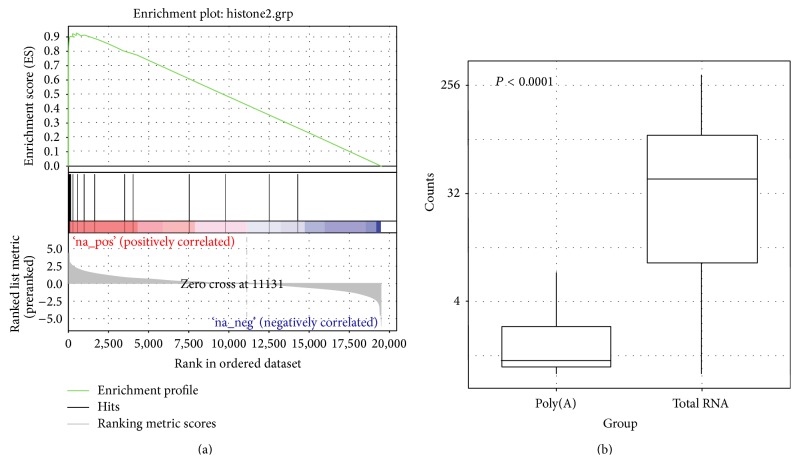
(a) Enrichment plot of histone-encoding genes from GSEA. Based on fold change ranked (total RNA versus poly(A)) gene list, histone-encoding genes were highly enriched (adjust *P* < 0.0001). (b) Normalized read count distribution of the 38 histone-encoding genes between poly(A) and total RNA libraries.

It has been shown that not all mRNAs necessarily contain a poly(A) tail at their 3′ ends [35]. For example, the mRNA that encodes histone proteins is nonpolyadenylated [36]. Another study has shown that a significant portion of the mRNA transcript has no poly(A) tail [37]. This can potentially explain why we observe more protein coding RNA detected by total RNA than the poly(A) method. To test this hypothesis, we searched through the ENSEMBL database and found 38 histone-encoding genes. We conducted enrichment analysis in GSEA using results from DESeq2 against the histone-encoding genes and found that our dataset was highly enriched (FDR < 0.0001) (Figure 5(a)). The expression value of the histone-encoding genes was clearly higher for total RNA library samples (Figure 5(b)). The GSEA showed that total RNA library samples captured histone-encoding genes at a much higher efficiency than the poly(A) library samples. Based on fold change results from DESeq2, there were 737 protein coding RNAs that have a log2 fold change greater than 2 (overexpressed in total RNA samples), which suggests that additional subsets of protein coding RNAs may be better captured using total RNA methods. To better categorize these potential subcategories of protein coding RNAs, we conducted GO analysis using WebGestalt (Figure S2) (Table 1). The top 10 subcategories of genes were found within all three big GO categories: biological process, molecular function, and cellular component. Eleven out of the 30 subcategories primarily consisted of histone-encoding genes. The other 19 subcategories were protein-DNA complex, chromatin, and so forth. No obvious pattern was recognizable. There were also 592 protein coding genes that were captured better by the poly(A) library samples (log2 fold change < −2). We also performed GO analysis on these genes (Figure S3) (Table 2). No clear gene pattern was detected.

## 4. Discussion

In this study, we examined the difference between the RNAs captured through poly(A) and total RNA libraries. Our study was also designed with several limitations. First, we were only able to collect two samples with sequencing data from both RNA libraries. The small sample size might limit our ability to identify true signals. Also, the sample type is limited to breast cancer cell lines. Other tissue types might behave differently.

Using sequencing data from two breast cancer cell lines captured using both libraries, we found that, in terms of expression level, both libraries were highly correlated and the correlation was the highest for protein coding RNAs. This suggests that both methods of RNA library construction are capable of generating consistent data for studying protein coding RNAs. For the three types of RNA we defined: protein coding RNA, lncRNA, and other RNAs; at all gene detection thresholds, total RNA library samples consistently identified more RNAs than poly(A) library samples which suggests that the total RNA library is capable of detecting additional RNA not detected by the poly(A) library. Through gene set enrichment analysis we were able to identify that histone-encoding genes were not captured efficiently by the poly(A) RNA library due to their lack of poly(A) tails. This finding is consistent with previous reports [36, 37]. Through gene ontology analysis we identified several additional subgroups of RNA which were better captured by the total RNA library. This could be explained in several ways. First, the results could be due to random variation, thus not holding any biological significance. Second, the poly(A) tails might have degraded prior to the construction of the poly(A) RNA library. Third, some unknown mechanisms may prevent proper capture of such RNAs through poly(A) identification.

Total RNA library construction costs around $150 more than a poly(A) library, but it allows the detection of additional RNAs. Whether the extra cost is justifiable should be decided during the experimental design stage of RNAseq study. If the goal is to study lncRNA, then it is better to use total RNA library; if the goal is to study protein coding RNAs, then total RNA library might not be necessary unless histone-encoding genes are of interest.

## Supplementary Material

Extracts of medicinal plants used in traditional Korean medicine investigated for cytotoxic activity towards cancer cells.

## Figures and Tables

**Table 1:** Gene ontology results of genes that are captured more by total RNA library.

Major category	Subcategory	Number of genes	Adjusted *P*
Biological process	Nucleosome assembly (histone)	30	4.81*E* − 16
	Protein-DNA complex assembly (histone)	33	6.27*E* − 16
	Chromatin assembly (histone)	30	2.58*E* − 15
	Protein-DNA complex subunit organization (histone)	33	9.36*E* − 15
	Nucleosome organization (histone)	30	2.36*E* − 14
	Chromatin assembly or disassembly (histone)	30	1.81*E* − 13
	DNA packaging (histone)	30	2.51*E* − 12
	DNA conformation change (histone)	30	7.46*E* − 10
	Cellular macromolecular complex assembly (histone)	38	6.50*E* − 03
	Detection of virus	3	7.30*E* − 03

Molecular function	Protein heterodimerization activity (histone)	32	5.00*E* − 04
	Ketosteroid monooxygenase activity	3	4.00*E* − 03
	Phenanthrene 9,10-monooxygenase activity	3	4.00*E* − 03
	cGMP binding	5	4.00*E* − 03
	Oxidoreductase activity	7	6.30*E* − 03
	Androsterone dehydrogenase activity	3	6.30*E* − 03
	Dehydrogenase activity	3	6.30*E* − 03
	Cyclic nucleotide binding	6	1.48*E* − 02
	N,N-Dimethylaniline monooxygenase activity	3	1.48*E* − 02
	Metal ion transmembrane transporter activity	25	2.83*E* − 02

Cellular component	Nucleosome (histone)	29	8.22*E* − 23
	Protein-DNA complex	30	4.93*E* − 17
	Chromatin	35	1.22*E* − 08
	Chromosomal part	40	1.00*E* − 04
	Chromosome	41	2.60*E* − 03
	Extracellular region part	59	1.33*E* − 02
	Axoneme	9	2.28*E* − 02
	Platelet dense tubular network membrane	3	6.32*E* − 02
	Platelet dense tubular network	3	1.15*E* − 01
	Desmosome	4	1.57*E* − 01

**Table 2:** Gene ontology results of genes that are captured more by poly(A) RNA library.

Major category	Subcategory	Number of genes	Adjusted *P*
Biological process	RNA metabolic process	174	1.30*E* − 03
	Nucleic acid metabolic process	188	4.50*E* − 03
	Cellular macromolecule metabolic process	260	5.30*E* − 03
	Positive regulation of cell development	17	5.50*E* − 03
	Transcription from RNA polymerase II promoter	75	5.50*E* − 03
	Cellular component organization	174	5.80*E* − 03
	Cellular component organization or biogenesis	177	6.90*E* − 03
	Positive regulation of cell morphogenesis	7	6.90*E* − 03
	Negative regulation of viral entry into host cell	3	7.00*E* − 03
	Regulation of transcription, DNA-dependent	126	7.00*E* − 03

Molecular function	Chromatin binding	27	1.00*E* − 03
	Protein binding	276	1.60*E* − 03
	Binding	400	3.90*E* − 02
	D-Erythro-sphingosine kinase activity	2	5.57*E* − 02
	Transcription cofactor activity	28	5.57*E* − 02
	Lipid kinase activity	3	5.57*E* − 02
	Sphinganine kinase activity	2	5.57*E* − 02
	Transcription factor binding transcription factor activity	28	6.34*E* − 02
	Nucleic acid binding	127	9.22*E* − 02
	Protein binding transcription factor activity	28	9.22*E* − 02

Cellular component	Nucleus	251	4.99*E* − 09
	Membrane-bounded organelle	349	1.44*E* − 06
	Intracellular membrane-bounded organelle	349	1.44*E* − 06
	Intracellular organelle	375	5.83*E* − 06
	Organelle	375	5.83*E* − 06
	Nuclear lumen	128	6.62*E* − 06
	Nuclear part	139	8.80*E* − 06
	Intracellular organelle lumen	144	3.83*E* − 05
	Organelle lumen	145	4.64*E* − 05
	Nucleoplasm	77	5.05*E* − 05
