# Supplementary material for: RNAseq by Total RNA Library Identifies Additional RNAs Compared to Poly(A) RNA Library
Source: Biomed Res Int. 2015 Oct 12;2015:862130. doi: 10.1155/2015/862130 (PMC4620295; doi:10.1155/2015/862130)
Supplement: Supplementary file 1 — Extracts of medicinal plants used in traditional Korean medicine investigated for cytotoxic activity towards cancer cells. [file 862130.f1.pdf]

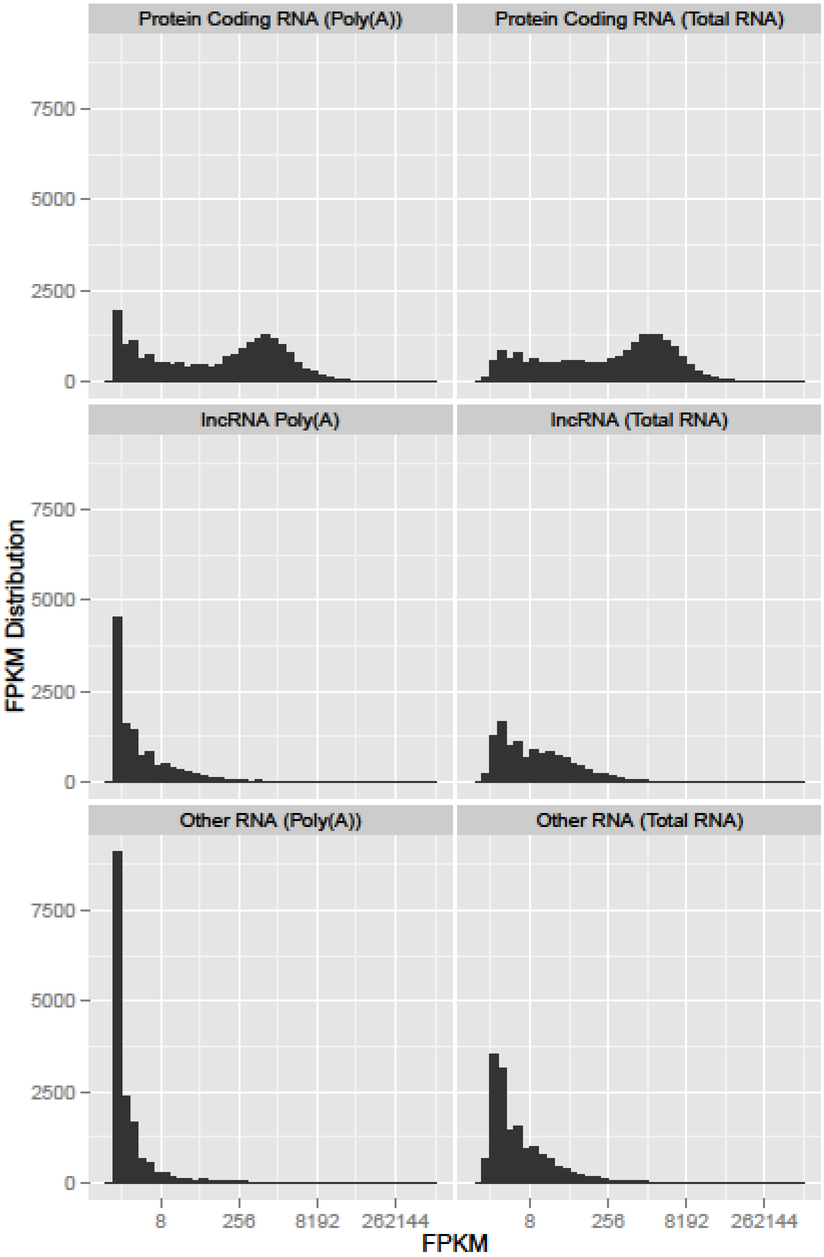

Figure S1. Normalized read count distribution of poly(A) and total RNA libraries for three types of RNA.

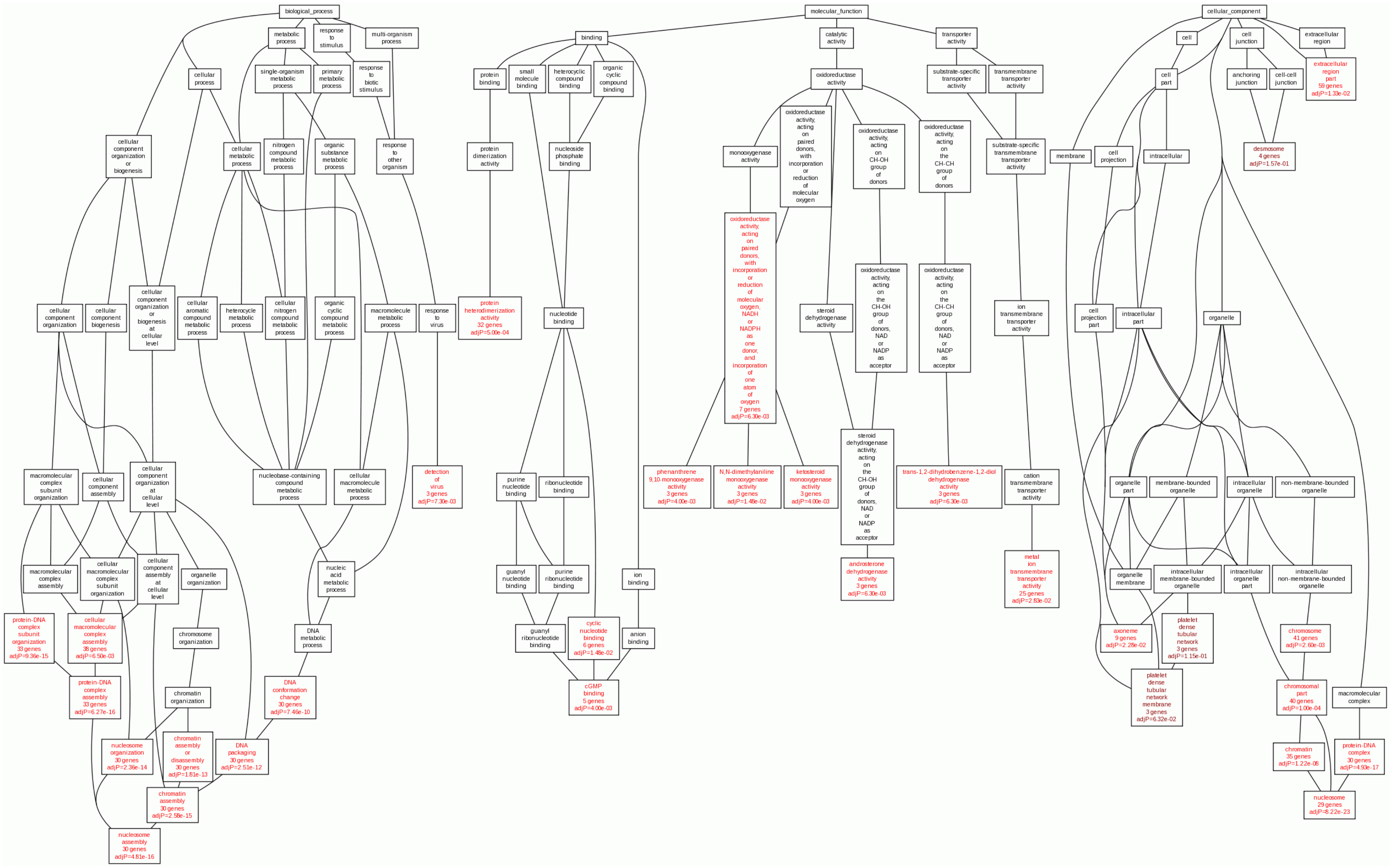

Figure S2. Gene ontology analysis results for protein coding RNAs identified more efficiently with total RNA libraries.

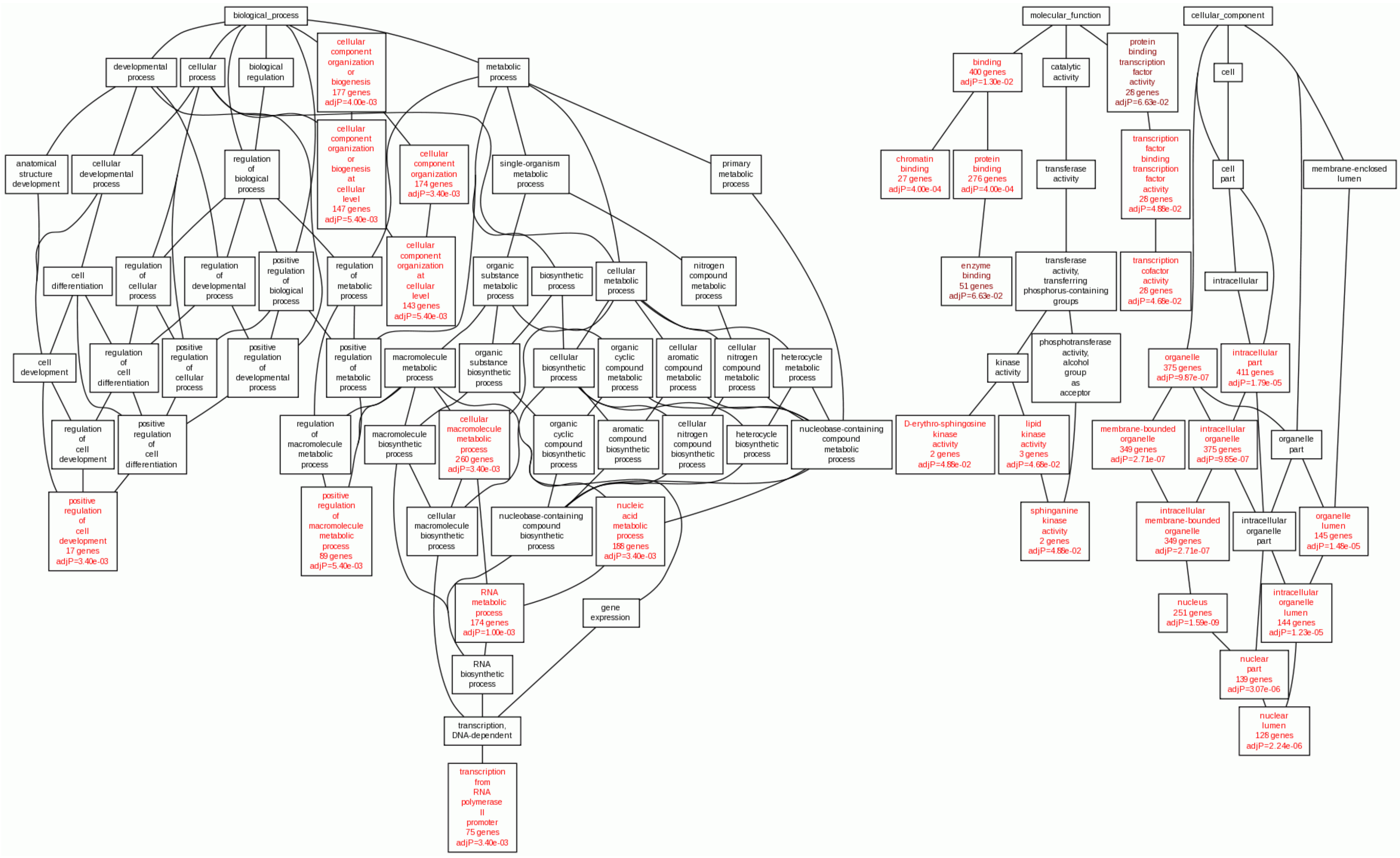

Figure S3. Gene ontology analysis results for protein coding RNAs identified more efficiently with poly(A) RNA libraries
